# Army Convalescent Homes

**Published:** 1899-02-25

**Authors:** 


### ARMY CONVALESCENT HOMES.

THE Parliamentary session could hardly have opened with a more satisfactory announcement—at least, to the medical and nursing professions—than the fact that nine Government convalescent barracks are at once

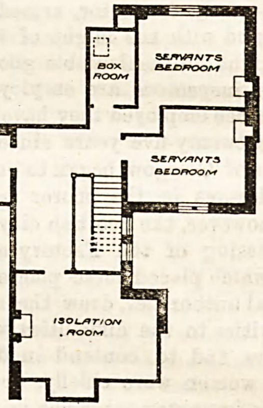

SECOND FLOOR PLAN

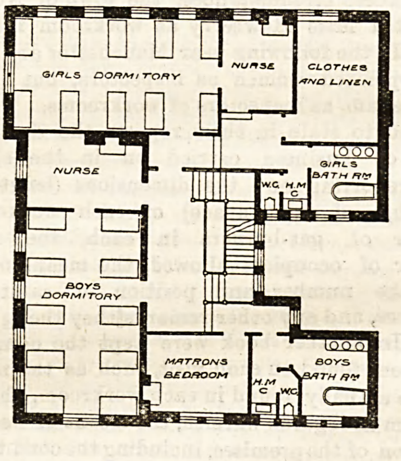

FIRST FLOOR PLAN

to be added to the Army Medical Service. The need of convalescent homes for soldiers has been agitated recently in the medical, lay, and military press, and several philanthropists had come forward with the intention of charitably providing homes which it was clearly the province of the State to institute.

Indeed, so recently as Saturday, the 18th inst., H.R.H. Princess Christian—whose interest in the welfare of the sick is whole-hearted and far-reaching—had summoned a special council meeting of the Soldiers' Help Society to consider how best convalescent homes for soldiers could be added to the practical programme of this society. Miss Annesley Kenealy, Captain Cecil Norton, M.P., and Lady Theodora Davidson, who have been for some time past actively interested in the cause of the convalescent soldier, were invited to attend the committee and lay their plans before the society.

The three delegates met the committee at half-past eleven a.m, on the 18th inst., and were pleased to be in a position to announce, on the authority of the War Office, that the Government had decided to take the initiative, and that nine convalescent homes were to be opened immediately. The meeting was unanimous in feeling that the case of the convalescent soldier rightly belongs to the State, though the Soldiers' Help Society held itself ready to assume the duty had the Government not announced its willingness to act in the matter. At present the soldier convalescents are to be accommodated in barracks situated in bracing and healthful situations, such as Sandown, Shorncliffe, Scarborough Aberdeen, Ayr, Pembroke Dock, and Yarmouth. The nine selected barracks are merely experimental. Later it is hoped to add special sunny convalescent wings to various barracks, and to choose some inland and mountain resorts for malarial, dysenteric, and gastric cases.

Now that the needs of the convalescent soldier have been officially recognised the Army Medical Service will doubtless carry out the scheme as thoroughly as the resources at their command allow.

Despite the proverbial injunction against examining the mouth of a gift horse, it is impossible to refrain from criticising one point of the convalescent programme. The rations provided at the proposed convalescent barracks are to be exactly the same as those served out to the strong, fit soldier on active duty. The convalescent from the enteric of India, the malaria of the West African Coast, and the fever of Malta will hardly be equal to the sturdy fare of the marching soldier. The dieting of the military invalid on the lines of the active soldier cannot be expected to prove an unqualified success, and this will doubtless appear later as the weak spot in the new system. The authorities of the War Office state that the order with regard to the diet has been made from motives of economy. Doubtless it will keep down the cost of the convalescent homes if these are dieted after the canteen pattern. But the economy is a doubtful one, since the man convalescing from serious diseases—and there will be many sick Tommys in the homes who are very sick indeed—can hardly be expected to make a very quick recovery on the "beef and bread" rations of a marching man. Time and experience, however, will rectify a matter of detail such as this. Meanwhile the gift to the sick soldier of a convalescent furlough, during which he will be relieved from all barrack duties, route marches, and fatigue, is indeed good. And the War Office confidently promises that the whole scheme can be carried through without adding any additional burden to the country beyond the cost of the soldiers' return railway tickets from quarters to convalescent home.

It is gratifying news, too, that six additional sisters are to be added to the Army Nursing Service, although it is not intended at present that the convalescent homes shall be under the superintendence of Army sisters. This step may be taken in time, but so far does not appear in the "special orders" which are shortly to be issued with regard to this new and important feature of the Army Medical Service.

